# FISSA: A neuropil decontamination toolbox for calcium imaging signals

**DOI:** 10.1038/s41598-018-21640-2

**Published:** 2018-02-22

**Authors:** Sander W. Keemink, Scott C. Lowe, Janelle M. P. Pakan, Evelyn Dylda, Mark C. W. van Rossum, Nathalie L. Rochefort

**Affiliations:** 1 Institute for Adaptive and Neural Computation, School of Informatics, University of Edinburgh, Edinburgh, EH8 9AB UK; 2 Bernstein Center Freiburg, Faculty of Biology, University of Freiburg, 79104 Freiburg, Germany; 3 Centre for Discovery Brain Sciences, Biomedical Sciences, University of Edinburgh, Edinburgh, EH8 9XD UK

## Abstract

*In vivo* calcium imaging has become a method of choice to image neuronal population activity throughout the nervous system. These experiments generate large sequences of images. Their analysis is computationally intensive and typically involves motion correction, image segmentation into regions of interest (ROIs), and extraction of fluorescence traces from each ROI. Out of focus fluorescence from surrounding neuropil and other cells can strongly contaminate the signal assigned to a given ROI. In this study, we introduce the FISSA toolbox (Fast Image Signal Separation Analysis) for neuropil decontamination. Given pre-defined ROIs, the FISSA toolbox automatically extracts the surrounding local neuropil and performs blind-source separation with non-negative matrix factorization. Using both simulated and *in vivo* data, we show that this toolbox performs similarly or better than existing published methods. FISSA requires only little RAM, and allows for fast processing of large datasets even on a standard laptop. The FISSA toolbox is available in Python, with an option for MATLAB format outputs, and can easily be integrated into existing workflows. It is available from Github and the standard Python repositories.

## Introduction

**Figure 1:**
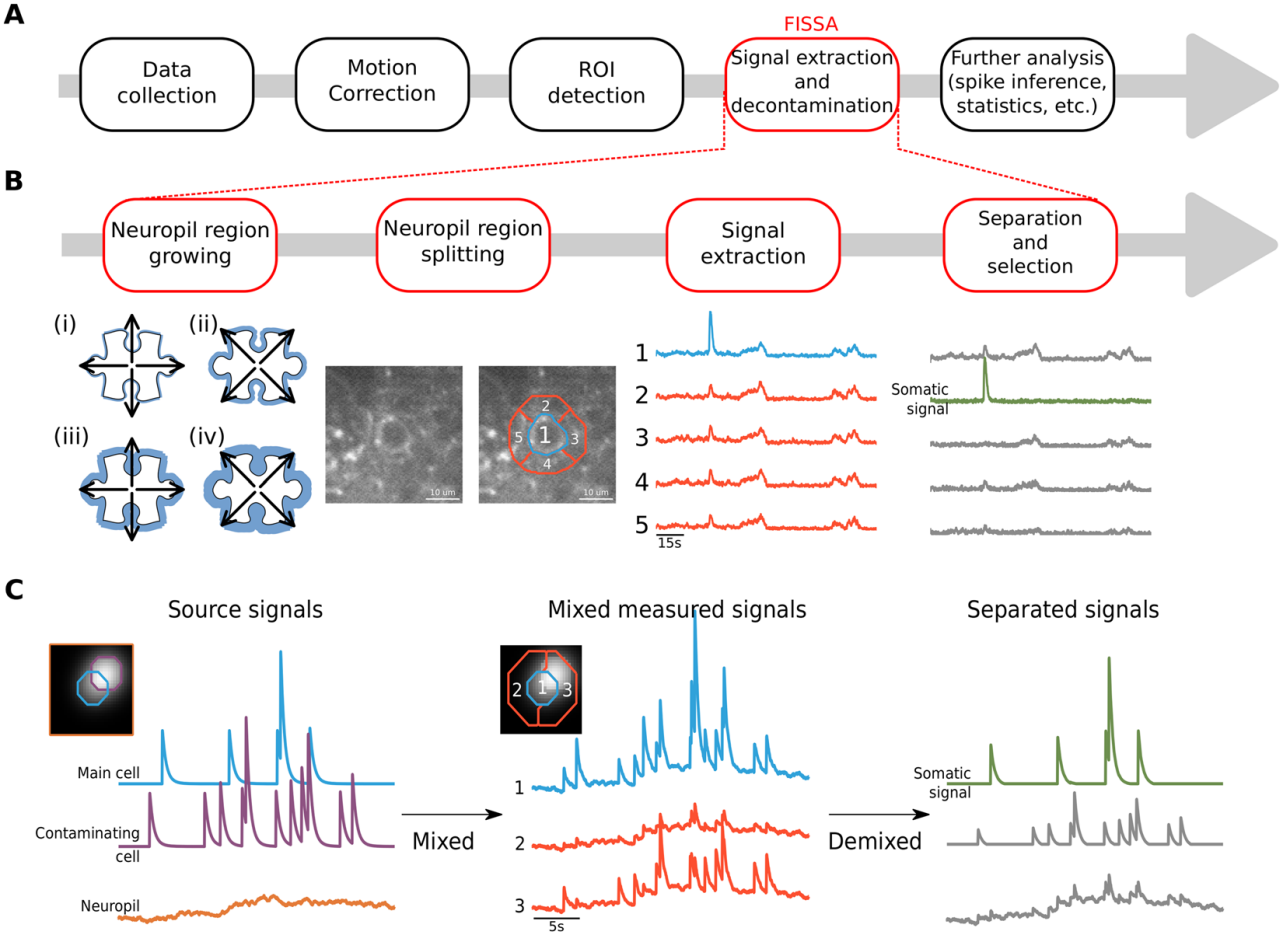
FISSA toolbox overview. (**A**) Schematic of a generic calcium imaging data analysis workflow. (**B**) Schematic of the four main steps of the FISSA toolbox workflow. Given a predefined region of interest (ROI), the local neuropil region is defined by expanding the ROI (white shape) alternately in the cardinal and diagonal directions (steps i to iv), until the surrounding area (blue area) is four times the ROI area. The resulting neuropil region is split into four subregions (regions 2, 3, 4, and 5). The signals from each of these regions are separated using non-negative matrix factorization (NMF), and the somatic signal is identified. (**C**) Schematic representation of blind source separation in FISSA. Left: Model of a somatic signal (blue) contaminated by two types of sources: the surrounding neuropil (orange), and an overlapping soma (purple). Middle: The measured signals in the somatic ROI (region 1) and in the surrounding neuropil (regions 2 and 3) will each be a mix of the three underlying source signals shown in the left panel. Right: NMF separates the mixed signals, recovering the original source signals. From these demixed components the one that best matches the measured ROI signal, relative to the neuropil regions, is identified as the somatic signal.

Recent developments in *in vivo* imaging and genetically-encoded calcium indicators enable monitoring the activity of hundreds to thousands of neurons in the brains of awake behaving rodents. The activity of sub-types of neurons can be directly related to the animal’s behaviour, over temporal scales from hundreds of milliseconds to several weeks^1–5^. Such imaging experiments produce large sequences of images, the analysis of which typically involves the following steps (Fig. 1A):

1. Correction of brain motion artefacts that lead to the misalignment of imaging frames from one time point to the next. For this step, open source software packages are available^6–13^.
2. Segmentation of the imaged field-of-view into regions of interest (ROI), typically containing individual neuronal soma or sub-cellular components (e.g. dendrites and spines). Images can be segmented either manually^4^, semi-automatically^3,14^, or automatically using either morphological criteria^8^ or activity based algorithms^8,9,15–18^.
3. Extraction of the fluorescence changes across time within each ROI. Because two-photon microscopes have an elongated point spread function along the Z-axis, the signals imaged in a given focal plane are contaminated by signals from above and below this plane. The fluorescence signal from a given region of interest is thus usually contaminated by signals from surrounding neurites (axons and dendrites) and sometimes nearby neuronal somata. Correcting for such out-of-focus contamination is particularly critical for experiments in which neuropil activity itself is modulated by the experimental protocol. Decontamination is the focus of the current paper.

Currently, two main approaches are used to correct for neuropil contamination, either by subtracting a neuropil signal from the somatic signal, or by using blind source separation methods. For subtraction, a neuropil region is defined around the region of interest (e.g. a soma), and the spatially averaged neuropil signal is subtracted from the somatic signal^3,14^. Since the neuropil signal is not spatially uniform^11,19^, using a local neuropil region is preferable over using a global neuropil signal. The subtraction method has the advantage of being fast and intuitive. However, subtraction can lead to negative signals when the neuropil signal is larger than the somatic signal, while in other cases it does not remove all contamination. As a consequence, the subtraction parameters (such as how much weight to give to the subtracted neuropil signal) have to be adjusted for each dataset or even for each cell type (sparse vs densely firing cells^14^), which makes standardization of this approach challenging.

A more recent class of decontamination methods is based on blind source separation. These methods aim at finding underlying signal sources from image sequences, typically using either Independent Component Analysis (ICA)^18,20^, Non-Negative Matrix Factorization (NMF)^15^, or model-based NMF^9,11^. These approaches are standardized and user-independent. They simultaneously estimate ROIs and their associated fluorescence signals, while also accounting for neuropil contamination. However, automated segmentation into somatic ROIs is not always reliable and hand labelling is often still necessary. In addition, in early versions of blind source separation methods, the neuropil was modelled as a one-dimensional signal shared by all pixels with different weights, which can lead to an artificial decrease of correlated somatic signals^9^. Finally, cell-detection methods are computationally intensive for large datasets. Thus, the ideal neuropil decontamination method would be standardized, fast, and work with both manually and automatically drawn ROIs.

For these reasons, we have developed the Fast Image Signal Separation Analysis (FISSA) toolbox for decontaminating calcium signals. FISSA defines a set of neuropil regions around pre-defined somatic ROIs (either from hand-labelling or from automatic detection algorithms), and uses NMF to separate the signals from these regions (Fig. 1B,C). We have tested this toolbox on both simulated and *in vivo* data, and our results show that FISSA performs either similarly or better than existing published methods. Additionally, since only a few signals need to be separated, FISSA is fast and requires only little RAM, so that it is usable on a standard computer or laptop.

## Results

### FISSA toolbox workflow

The goal of the FISSA toolbox is to remove contamination from the ROI signals. As a consequence of the limited resolution of *in vivo* imaging methods, especially axially^21^, the signal measured from a given region of interest in a single focal plane is in fact a mixture of signals originating from this ROI as well as from a surrounding volume (Fig. 1C). This volume includes mostly neurites (axons and dendrites) of other cells, and sometimes other somata. To demix these signals, we use the fact that the signals (photon counts) are always positive, and assume that the mixing of the different signals is linear and additive. A method of choice for demixing signals under these assumptions is non-negative matrix factorization^22,23^, which separates signals by estimating a set of positive signals that best explains the observed mixed signals.

**Figure 2:**
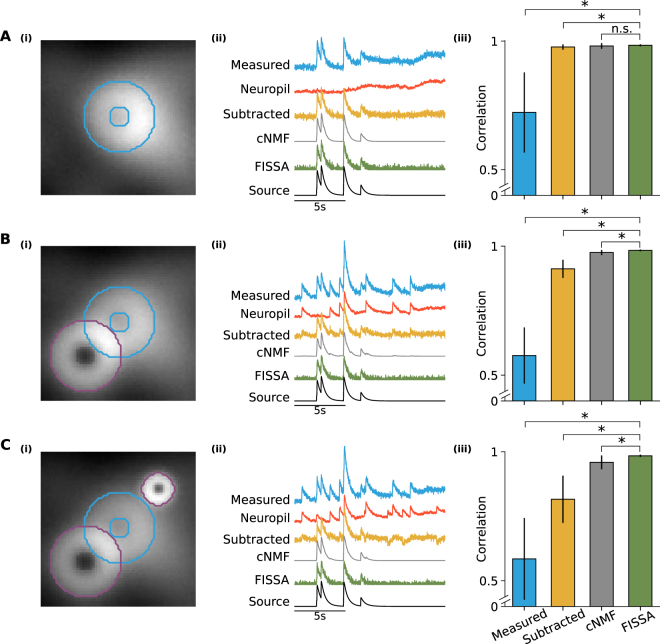
Comparison of FISSA performance with other decontamination methods on simulated calcium imaging data. Rows **A**, **B**, and **C** illustrate three cases with increasing levels of contamination. (**A**)(i) Image of a region containing a single doughnut-shaped cell firing at 0.5 Hz and a fluctuating neuropil background. The image is an average across all 12000 frames (120 s). The ROI is indicated in blue. (ii) Example fluorescence traces for the blue ROI indicated in panel A(i): average measured signal of the ROI (blue trace, ‘Measured’), the average surrounding neuropil signal (red trace), and the signals after neuropil decontamination by three different methods (subtraction in yellow, cNMF in grey, and FISSA in green). The uncontaminated source signal (as defined by the simulation) is shown in black. The cNMF trace has a lower noise level as the cNMF algorithm also includes smoothing. (iii) Average Pearson correlations between the ROI source signal and the extracted ROI signal: before decontamination (first column, ‘Measured’, 0.723), and after each decontamination method (neuropil subtraction, cNMF, and FISSA; 0.977, 0.981, and 0.984 respectively). Error bars indicate standard deviation. *p < 0.05; **p < 0.005; n.s.: not significant (p > 0.05); Wilcoxon signed-rank test, *n* = 10 simulations of 120 s each (with different background neuropil signals, spike times, and photon noise). FISSA vs: measured *p* = 0.0033, subtraction *p* = 0.0409, cNMF *p* = 0.0911. (**B**) As panel A, but the blue ROI is additionally contaminated by an overlapping cell firing at 0.3 Hz (indicated by the purple outline). Average values for the four methods are 0.576, 0.912, 0.975, and 0.984 respectively. FISSA vs: measured *p* = 0.0033, subtraction *p* = 0.0033, cNMF *p* = 0.0033. (**C**) As panel B, but an additional bright localized signal firing at 0.3 Hz was added (smaller purple outline). Average values for the four methods are 0.585, 0.816, 0.959, and 0.984 respectively. FISSA vs: measured *p* = 0.0051, subtraction *p* = 0.0051, cNMF *p* = 0.0051.

The FISSA toolbox relies on user-defined ROIs that can be imported either from ImageJ or defined as standard Python arrays. For each ROI, FISSA first defines a neuropil region by expanding the shape of each ROI by a fixed amount (Fig. 1B and Methods). The neuropil area is defined as the expanded shape, excluding the original ROI. Next, the neuropil region is divided into subregions of equal area. By default, FISSA defines four subregions each with the same area as the ROI; performance did not improve with more subregions (Fig. 2E).

The signals from each region (the four subregions of the neuropil and the somatic ROI) are then separated using NMF. The NMF method returns how strongly each separated signal was present in each subregion and ROI. Using these estimates, the separated signals are scaled and sorted by relative presence in the ROI compared to the surrounding subregions (see Methods). The signal with the strongest relative presence is taken as the extracted somatic signal (Fig. 1C).

Note that FISSA separates the raw fluorescence signals. The calculation of the relative change in fluorescence (Δ*f*/*f*_0_) can be done afterwards (see Methods). The extracted and decontaminated signals can be accessed in Python or saved in MATLAB format.

### FISSA performance on simulated calcium imaging data

We first used simulated data to evaluate our approach. We modelled a set of neurons with Poisson firing statistics and calcium indicator dynamics based on GCaMP6f (see Methods). In the model, each cell had a well defined spatial structure, but its signal bled into the surrounding region and cells could overlap. A smoothly fluctuating global neuropil signal was included to model background fluctuations. A given pixel might thus contain the global neuropil contamination, and one or more cell signals. Finally, to model photon emission, the calcium indicator signal at each pixel was simulated by Poisson shot noise.

We evaluated the performance of different decontamination methods on three cases with increasing contamination (Fig. 2). We compared the performance of three decontamination methods: 1) subtraction of the local surrounding neuropil signal^3,14^, 2) a cell detection and signal separation method including neuropil decontamination called constrained NMF (cNMF^9^), and 3) FISSA extraction. To quantify the performance of each method we calculated the Pearson correlation between the extracted signals and the ROI source signal, with the extracted signals low-pass filtered at 5 Hz to minimize differences due to high frequency noise.

We first considered a single cell whose somatic signal was only contaminated by neuropil fluctuations (Fig. 2A). All three methods successfully decontaminated the ROI signal, resulting in a high correlation between the corrected signal and the ROI source signal (Fig. 2Aiii).

Next, we modelled the same soma but added a partially overlapping neuron (Fig. 2B). The additional contaminating calcium transients reduced the correlation between the measured and the ROI source signal (Fig. 2Biii). Whilst subtracting the neuropil signal still removed slow fluctuations, it did not fully remove the extra transients, resulting in a lower correlation than the other two methods. cNMF and FISSA removed both the background fluctuations and the contaminating transients equally well, while preserving the true transients.

Finally, we added a second non-overlapping signal source with a strong, localized calcium response (Fig. 2C), leading to a neuropil signal with additional large calcium transients (Fig. 2Cii). The subtraction method led to negative transients, and the correlation between its signal and the source signal was lower than for the other two methods. FISSA and cNMF both resulted in very high correlations, with FISSA’s being slightly but significantly higher (*p* = 0.0051, Fig. 2Ciii).

**Figure 3:**
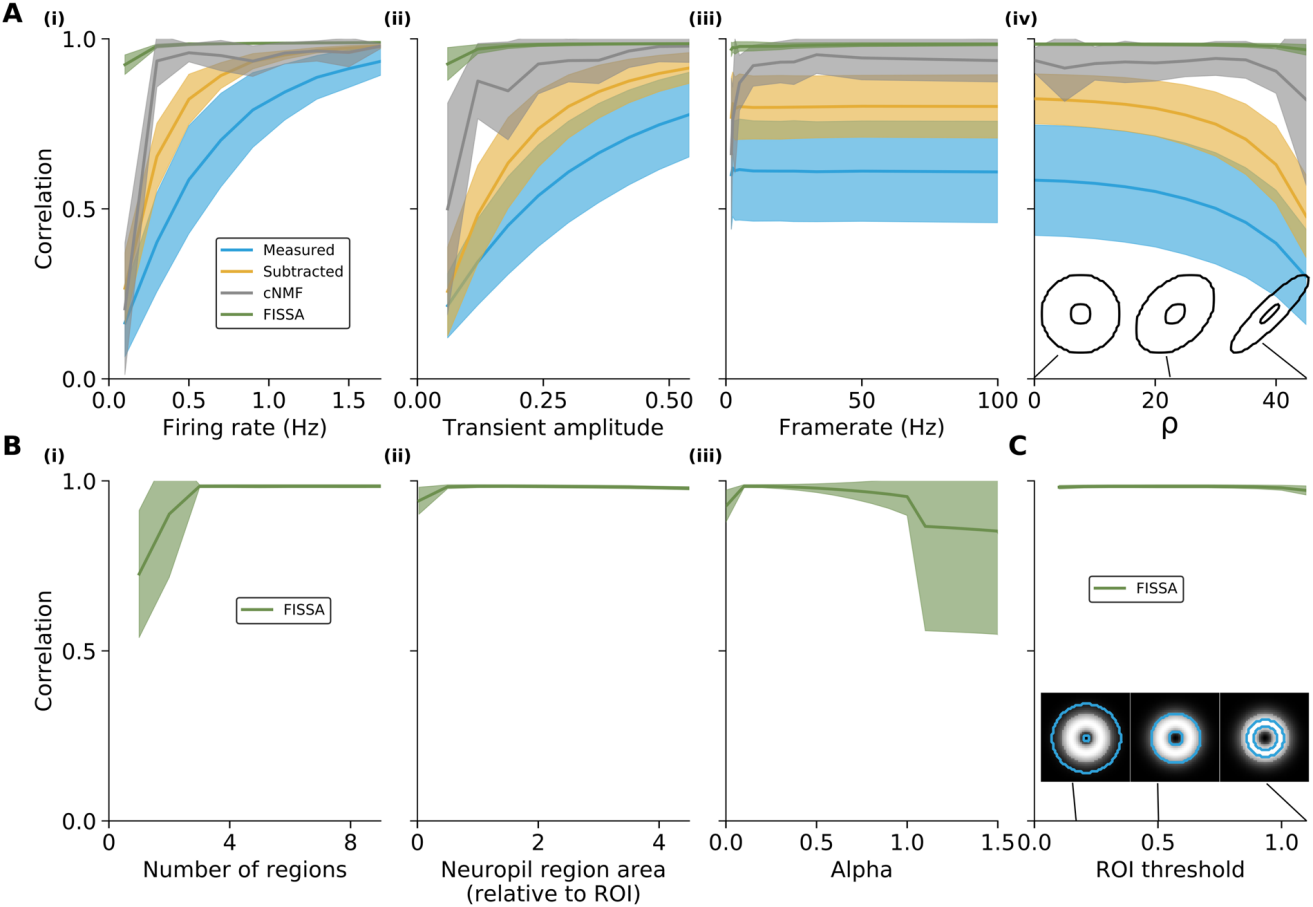
FISSA performance for different simulated data parameters and user-adjustable FISSA parameters, for the signal of the cell of interest from the case in Fig. 2C. (**A**) Correlations between the source and extracted signals before neuropil decontamination (‘Measured’) and after each decontamination method (neuropil subtraction, cNMF, and FISSA) for different simulation parameters. (i) Correlations for changes in the firing rate of the cell of interest (0.1 to 1.7 Hz, with steps of 0.2 Hz, default is 0.3 Hz). (ii) Correlations for changes in calcium transient magnitude (parameter *A*, see Eq. 9. 0.06 to 0.54 with steps of 0.06, default is 0.3). (iii) Correlations for changes in imaging framerate (downsampling of 100 Hz initial data to lower frame rates: by 50, 40, 30, 20, 10, 5, 4, 3, 2, and 1). (iv) Correlations for changes in cell shape, by changing the parameter *ρ*, see Eq. 10 (steps of 5 from 0 to 45, default is 0). The insets show example cell shapes for different *ρ* values. (**B**) Correlations between the source and extracted signals against different user-adjustable FISSA parameters (x-axes). (i) Number of neuropil subregions while keeping the total area constant, at four times the ROI area. (ii) Area of the neuropil subregions relative to the central ROI (0.025 for the smallest area, steps of 0.5 from 0.5 to 4), for four subregions. (iii) NMF parameter *α* (with steps of 0.1). (**C**) The effect of suboptimal ROI selection, by varying the threshold at which a cell mask is defined (Parameter ${T}_{{\mathtt{mask}}}$ in Eq. 14. 0.1 to 1.1 with steps of 0.1, default is 0.5). The insets show the same central cell with the outlines of the mask used for three example thresholds. All panels show the average values over 10 simulations; shaded areas indicate standard error.

We then tested whether the results were consistent across a broader range of simulation parameters (Fig. 3A). While keeping the firing rates of the other cells the same, we varied the firing rates of the cell of interest (panel Ai), its spike transient amplitude (panel Aii), and the imaging frame-rate (panel Aiii). The correlations generally decreased for all methods as the signal-to-noise ratio decreases (through lower firing rates or calcium transient amplitudes). FISSA maintained the highest correlation across all parameter changes, and in particular at low signal-to-noise ratios performed better than other methods. In some cases, the difference between cNMF and FISSA results does not reflect the performance of the signal separation per se but rather shows the limit of the automatic detection method of cNMF. In cases of low signal amplitude or firing rate, cNMF may simply not detect the cell of interest. As a consequence, since there is no segmentation and thus no signal, the correlation with the source signal is very low. However, for the same reasons, a cell with very low firing rates might also not be detected manually. We then tested whether FISSA performed equally well when taking only a subset of the data. Our results show similar performance when downsampling a 120 s data set from 100 Hz to lower frame rates (Fig. 3Aiii). Finally, the shape of the cell of interest, such as more elongated shapes, also did not substantially affect the performance (Fig. 3Aiv).

FISSA has a number of user-adjustable parameters: the number of neuropil regions, the area of the neuropil subregions, and the NMF parameter *α* which promotes sparseness of the source separation. Performance is robust across parameter values (Fig. 3B), as long as the number of neuropil regions is larger than 3 (the default is 4), for a neuropil subregion area at least half of the original ROI’s area (default is the same size as the ROI), and an *α* between 0.1 and 0.5 (the default is 0.1). Finally, we tested the influence of the ROI’s size relative to the cell of interest by varying the threshold used to define the ROI, while keeping the simulated cell shape constant (Fig. 3C). The results show that FISSA performance remains stable for a broad range of ROI sizes, either larger or smaller than the actual cell’s shape (Fig. 3C). This robustness of FISSA is a useful property when using hand-labelled ROIs that may be larger or smaller than the actual cell body.

### FISSA performance on *in vivo* calcium imaging data

We next tested FISSA on *in vivo* two-photon calcium imaging data of GCamP6-labelled layer 2/3 neurons in the mouse primary visual cortex. We used a publicly available dataset which contains simultaneous calcium imaging of GCamP6-labelled neurons and simultaneous cell-attached electrophysiological recordings^3,24^, allowing for a direct comparison between the extracted calcium signals and the recorded spikes. To quantify performance we calculated the Pearson correlation between the calcium signals estimated by each method, and the predicted calcium transients inferred from the recorded spikes.

**Figure 4:**
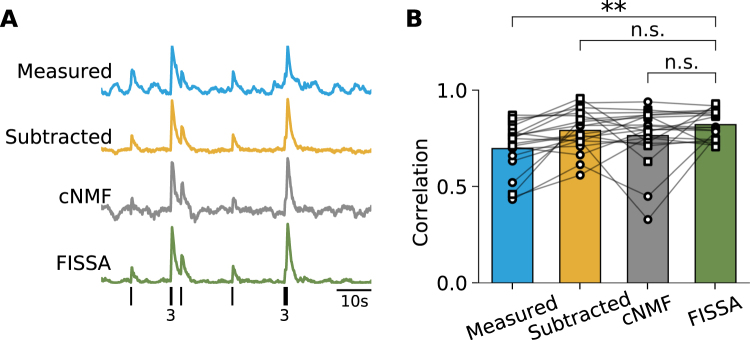
Comparison of neuropil decontamination methods on *in vivo* two-photon calcium imaging data from layer 2/3 neurons in mouse primary visual cortex (data from^3,24^). (**A**) Example measured and decontaminated traces of fluorescence changes for cell 20120627_cell4 (frames 14400 to 19200). All traces were low-pass filtered at 5 Hz. The black ticks on the bottom row indicate the spikes measured electrophysiologically, with the number of overlapping spikes indicated below. (**B**) Correlations between the calcium transients inferred from the recorded spikes (see Methods), and the calcium signals estimated by each method. The data points correspond to GCaMP6s (circles) and GCaMP6f (squares) cells from the dataset^3,24^. **p < 0.005; n.s.: not significant (p > 0.05); Wilcoxon signed-rank test, *n* = 20 cells, FISSA vs measured: *p* = 0.0006, FISSA vs subtraction *p* = 0.2959, FISSA vs cNMF *p* = 0.4330.

FISSA successfully decontaminated the somatic signal of 20 cells tested: the results showed significantly improved correlation values after FISSA compared to raw data (*p* = 0.0006, Fig. 4B, ‘Measured’ vs ‘FISSA’). On this dataset, the results obtained with the cNMF and subtraction methods were not significantly different from those obtained with FISSA (FISSA vs subtraction *p* = 0.2959, FISSA vs cNMF *p* = 0.4330, Fig. 4B). This difference in results, compared to the simulated data in Fig. 2, is due to the relatively sparse labelling leading to a low level of contamination with few overlapping labelled structures in the field of view. As such, the main contamination source consists of background fluctuations, for which all three decontamination methods work well. However, cNMF did result in low correlation values for a small subset of cells. This can be partly explained by the high optical magnification used for the dataset (roughly one to three cells per 30 *μ*m by 30 *μ*m field of view, at 256 by 256 pixels), while cNMF was designed to be applied to a large field of view with hundreds to thousands of cells. For some cells, the ROIs that cNMF extracted did not fully match the outline of the soma and not all contamination was successfully removed (e.g. grey trace, Fig. 4A).

Finally, we compared the different methods for neuropil decontamination in a dataset of *in vivo* two-photon calcium imaging of layer 2/3 neurons in the primary visual cortex (V1) of awake behaving mice. All neurons were labelled through local injection of adeno-associated viruses (AAV1.Syn.GCaMP6f.WPRE.SV40) in V1^4^. After 2–3 weeks of expression, running speed of the animal and GCaMP6f signals were recorded simultaneously during the presentation of drifting gratings. It is known that visual responses of layer 2/3 neurons in V1 are modulated by locomotion^4,25–29^. We compared the effect of locomotion on single neuron activity in this dataset before and after neuropil decontamination. The effect of locomotion was quantified for each neuron by the locomotion modulation index (LMI)^4^, which calculates the normalized difference between the mean change in fluorescence (Δ*f*/*f*_0_) during locomotion (*R*_*L*_) and stationary (*R*_*s*_) periods, as ${\mathtt{LMI}}=({R}_{L}-{R}_{s})/({R}_{L}+{R}_{s})$.

**Figure 5:**
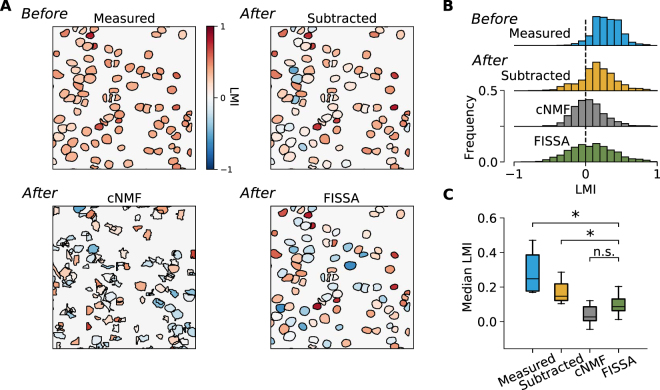
Impact of neuropil decontamination on neuronal responses measured with two-photon calcium imaging, in V1 layer 2/3 of awake behaving mice. Neurons were labelled with GCaMP6f^4^. The effect of locomotion was quantified for each neuron by the locomotion modulation index (LMI). (**A**) An example field of view with somatic ROIs coloured by the LMI value obtained either before neuropil decontamination (‘Measured’) or after decontamination. The ROIs for the measured, subtraction, and FISSA methods are identical and were defined by hand. For the cNMF method the ROIs are presented as detected by the algorithm. LMI values were calculated for periods of visual stimulation with oriented gratings (10 to 20 trials per field of view, 60 s/trial). (**B**) The distributions of LMI values across all layer 2/3 cells in eight mice, before (‘Measured’) and after each decontamination method. (**C**) The distributions of median LMI values for the eight mice, before and after decontamination. *p < 0.05; n.s.: not significant (p > 0.05); Wilcoxon signed-rank test, *n* = 8 mice, FISSA vs measured: *p* = 0.0117, FISSA vs subtraction *p* = 0.0117, FISSA vs cNMF *p* = 0.0687, subtraction vs cNMF *p* = 0.0687.

Our results show that before neuropil decontamination almost all cells in this dataset displayed positive LMI (Fig. 5, median LMI 0.29), indicating an increase of activity during locomotion. However, after neuropil decontamination by any of the three methods, LMI values strongly decreased (median LMI was 0.16, 0.04, and 0.09 using neuropil subtraction, cNMF, and FISSA, respectively). These results are in agreement with previous electrophysiological experiments which reported that 20–50% of neurons with visual responses are positively modulated by locomotion in mouse V1^28,30^. The LMI values obtained with FISSA were not significantly different from those obtained with cNMF (*p* = 0.0687). However, in this dataset, the subtraction method led to significantly higher LMI values than those obtained after FISSA and cNMF (*p* = 0.0117).

Altogether, these results show that correction for neuropil contamination is critical for the analysis of two-photon calcium imaging data, especially for datasets in which the surrounding GCaMP6-labelled neuropil is itself modulated by the experimental conditions (e.g. by sensory stimuli or by the animal’s behaviour). In addition, the results obtained with both simulated data and *in vivo* two-photon calcium imaging datasets indicate that FISSA performs either similarly or better than other published methods for neuropil decontamination.

### FISSA computational resources and integration into existing workflows

FISSA is freely available at https://github.com/rochefort-lab/fissa. The toolbox can be applied to an existing dataset in just a few lines of code:
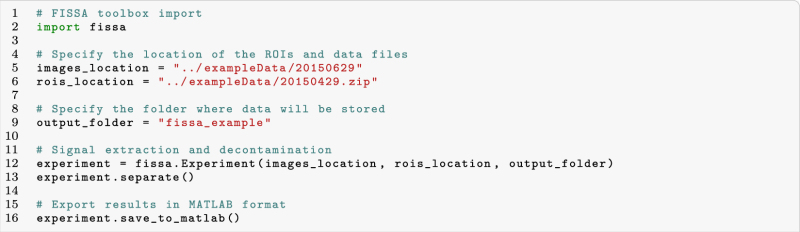


First, the user defines:

- The imaging data, either as a directory containing tiff files of the acquired images or standard Numpy arrays (Python format that can be generated from other data formats).
- The regions of interest (zip files of ROIs defined in ImageJ or Numpy arrays).
- The results folder where extracted and processed data will be stored.

After this step, only two lines of code are necessary to run the full FISSA analysis pipeline: from neuropil region definition to signal separation and selection. The results can then be accessed within Python or saved in MATLAB format. In the GitHub repository we provide example scripts which demonstrate how to integrate FISSA with existing published workflows such as SIMA^8^ and cNMF^9^.

By default, tiff files are fully loaded into memory before signal extraction. For large tiff files, there is an option to load them frame-by-frame to reduce memory usage. For other formats, there is also the option to define a custom data-loading script.

Since FISSA only has to separate a small number of signals, it can quickly separate signals across large datasets. For example, to extract isolated signals from 40 cells within a 600 × 600 pixel field of view over 2400 frames, FISSA only takes 40 seconds. This scales sub-linearly with the number of frames, such that for 30000 frames FISSA takes 60 seconds (for these tests FISSA was run on a workstation running Ubuntu 17.04 with a six core Intel Core i7-6800 K CPU@3.40 GHz). FISSA is thus well suited for processing large datasets with long imaging periods. However, as opposed to other methods (cNMF^9^, suite2P^11^), FISSA does not automatically segment the images into defined ROIs; therefore, the total time for data processing also depends on the method used for defining ROIs as well as on the data loading time.

## Discussion

We have developed a fast and easy to use toolbox (FISSA) for neuropil decontamination in calcium imaging datasets. The results obtained with both simulated and *in vivo* two-photon calcium imaging datasets indicate that FISSA performs either similarly or better than previously published methods for neuropil decontamination of calcium signals. In addition, FISSA presents a number of advantages. First, unlike subtraction methods, it provides a standardized, user-independent approach that removes multiple sources of contamination without leading to negative signal artefacts. Other methods based on blind-source separation do provide a standardized approach but require more computational resources and can be very slow to run on large datasets (although recent developments have improved the running time of this type of method^11,31^).

A further advantage of FISSA is that it uses minimal computational resources and can thus be used on a standard laptop even for large datasets. However, the total analysis time will also depend on the ROI detection method. FISSA does not include an automatic cell detection algorithm: ROIs must be defined beforehand, either manually or through a third-party algorithm. Separating ROI detection and signal extraction can be an advantage for experimental data in which automatic cell detection methods are not sufficiently accurate.

Finally, FISSA’s main assumptions are that measured calcium signals are positive and mix both linearly and additively. This is in contrast to other methods, that make specific assumptions about the calcium dynamics in terms of noise level and time scales^9,11^. Thus, FISSA is more generally applicable across various experimental conditions which may include different fluorescent indicators (such as synthetic dyes or other types of protein-based sensors) as well as other imaging methods (both *in vitro* and *in vivo*). Because the FISSA toolbox can be adapted to different formats of imaging data and regions of interest, it can easily be integrated into existing data analysis workflows.

## Methods

### FISSA algorithm

#### Neuropil subregions definition

FISSA uses predefined ROIs, obtained from either manual or automatic segmentation. The surrounding region is automatically defined by expanding the shape of each ROI alternately in the cardinal and diagonal directions (Fig. 1B). Next, the neuropil region is divided into *N* equal area subregions, by taking the polar coordinates relative to the ROI centre and taking the $\tfrac{1}{N}^{\prime} {\rm{th}}$ fraction for each subregion. By default, the total surrounding region is expanded until its area is *N* times the area of the central ROI (such that each subregion has the same area as the ROI). For the examples in this paper we set *N* = 4, as performance saturated at this *N* (Fig. 3Bi).

#### Non-Negative Matrix Factorization implementation

We assume that the spatially averaged signal in a given ROI, ${{\bf{f}}}_{{\mathtt{measured}}}(t)$, is given by a linear mixing of a set of underlying source signals

1 $${{\bf{f}}}_{{\mathtt{measured}}}(t)=W\,{{\bf{f}}}_{{\mathtt{source}}}(t),$$

where ${{\bf{f}}}_{{\mathtt{source}}}(t)$ is the set of source signals, and *W* is the mixing matrix. Blind source separation techniques estimate the mixing matrix *V* and separated sources ${{\bf{f}}}_{{\mathtt{sep}}}(t)$ such that

2 $${{\bf{f}}}_{{\mathtt{measured}}}(t)\approx V\,{{\bf{f}}}_{{\mathtt{sep}}}(t\mathrm{).}$$

For FISSA, we define ${{\bf{f}}}_{{\mathtt{measured}}}(t)$ as the *N* + 1 signals from the central ROI and the neuropil subregions, *V* is the *N* + 1 by *N* + 1 mixing matrix, and ${{\bf{f}}}_{{\mathtt{sep}}}(t)$ are the *N* + 1 extracted signals. The separation thus assumes the number of output signals is the same as the number of input signals (measured N + 1 signals). If there are fewer than *N* + 1 source signals being mixed (as, for example, in Fig. 2), we did not find this to have a negative impact on performance. The main point is that the number of output signals should not be lower than the number of signal sources. However, in physiological data, the number of signal sources is unknown. For the data sets we have used (*in vivo* data from local labelling of cortical neurons in V1), the number of N + 1 (corresponding to 5 regions) gave robust results. However, the option of changing the number of separated signals can be useful in case alternative data sets are likely to include more signal sources.

FISSA performs the blind source separation with Non-Negative Matrix Factorization (NMF), using the implementation in the scikit-learn toolbox^32^. There is also the option in the FISSA toolbox to use Independent Component Analysis (ICA) instead of NMF. ICA relies on the fact that the distribution of the sum of random variables will be more Gaussian than the individual components. Thus, by finding the most non-Gaussian projections, the sources can be identified. ICA is faster than NMF, but the resulting source signals or mixing coefficients can be negative, which can sometimes lead to negative extracted signals similar to the subtraction method (see Supplementary Fig. 1). NMF makes the stronger assumption that all signals and mixing coefficients are strictly non-negative, a property which is true for calcium imaging data. We therefore recommend using the NMF method, which was used for all results presented in this paper.

To perform NMF the temporal signals over time ${{\bf{f}}}_{{\mathtt{measured}}}(t)$ and ${{\bf{f}}}_{{\mathtt{sep}}}(t)$ are written as matrices with components

3 $${F}_{i,t}={f}_{i}(t\mathrm{).}$$

The NMF algorithm then minimizes an objective *E* in alternating steps with respect to *V* and ${F}_{{\mathtt{sep}}}$, until *E* reaches a target threshold^22,33^. The objective is the total squared difference between the measured signals and the estimated signals ${F}_{{\mathtt{measured}}}$, plus additional norms that encourage a sparse solution

4 $$\begin{array}{rcl}E & = & \frac{1}{2}\parallel {F}_{{\mathtt{measured}}}-V\,{F}_{{\mathtt{sep}}}{\parallel }_{{\mathtt{Fr}}{{\mathtt{o}}}^{2}}\\  &  & +\,\alpha \,{l}_{1{\mathtt{ratio}}}\parallel V{\parallel }_{1}+\alpha \,{l}_{1{\mathtt{ratio}}}\parallel {F}_{{\mathtt{sep}}}{\parallel }_{1}\\  &  & +\,\alpha \,\mathrm{(1}-{l}_{1{\mathtt{ratio}}})\parallel V{\parallel }_{{\mathtt{Fr}}{{\mathtt{o}}}^{2}}+\alpha \,\mathrm{(1}-{l}_{1{\mathtt{ratio}}})\parallel {F}_{{\mathtt{sep}}}{\parallel }_{{\mathtt{Fr}}{{\mathtt{o}}}^{2}}\end{array}$$

where the Frobenius norm is given by $\parallel A{\parallel }_{{\mathtt{Fr}}{{\mathtt{o}}}^{2}}=\frac{1}{2}\,{\sum }_{i,j}\,{A}_{ij}^{2}$ and the element-wise L1 norm is given by $\parallel A{\parallel }_{1}={\sum }_{i,j}\,|{A}_{ij}|$. ${l}_{1{\mathtt{ratio}}}$ determines the ratio between the Frobenius norm and the element-wise norm. *α* is the sparseness regularizer on both the mixing matrix *V* and the separated signals ${F}_{{\mathtt{sep}}}$. We set *α* = 0.1 and ${l}_{{\rm{1}}{\mathtt{ratio}}}=0.5$. Finally, both the separated signals and the mixing matrix are constrained to be non-negative. Initialization of both the estimated mixing and separated matrix is done by non-negative double singular value decomposition^34^.

#### Signal selection

Blind source separation returns a set of separated signals, but it does not indicate which one corresponds to the somatic signal and which ones are the contaminating signals. The estimated mixing matrix *V* provides the weight with which each identified source signal contributes to the mixed signals. Each row in the mixing matrix represents how strongly each of the estimated underlying signals is present in the measured signal. For each signal, we rate its relative presence in the central ROI by normalising the weights across each column of the mixing matrix

5 $${v^{\prime} }_{ij}=\frac{{v}_{ij}}{{\sum }_{i^{\prime} }\,{v}_{i^{\prime} j}}\mathrm{.}$$

The values ${v^{\prime} }_{ij}$ represent how strongly each signal is present in each region, relative to its average across all regions. The estimated somatic signal is then given by the signal for which ${v^{\prime} }_{0j}$ is the highest, multiplied by its contribution to the measured ROI signal

6 $${f}_{{\mathtt{est}}}(t)={v}_{0{j}_{{\mathtt{\max }}}}\,{f}_{{\mathtt{sep}}}^{{j}_{{\mathtt{\max }}}}(t),$$

where ${f}_{{\mathtt{sep}}}^{j}(t)$ is the *j*-th signal as separated by blind source separation, and

7 $${j}_{{\mathtt{\max }}}={\rm{\arg }}\,\mathop{{\rm{\max }}}\limits_{j}\,{v^{\prime} }_{0j}.$$

Thus, in order to find the signal that corresponds to the somatic signal, we assume that the somatic signal is more strongly represented in the central ROI, compared to the neuropil subregions (otherwise it is unlikely that it would have been chosen as the ROI). For example, the amplitude of the neuropil signal in the somatic ROI might be higher than the somatic signal itself, but this high neuropil signal will also be strongly present in the surrounding subregions. The somatic signal however, will be the one that is mostly present in the somatic ROI, but not in the other subregions.

#### Baseline

For Fig. 5, to calculate Δ*f*/*f*_0_, *f*_0_ was estimated as the 5^th^ percentile of the 1Hz low-pass filtered trace. For all extracted traces the *f*_0_ of the non-corrected trace was used.

#### Multiple trials

In the case of several discrete recording sessions of the same cells, by default FISSA concatenates the trials together. This is done to ensure that the signal extracted for a given trial does not suddenly change across trials (e.g. if a cell is silent during one trial).

#### Software

The code is implemented in Python 2.7, using the Numpy 1.7, SciPy 0.12, Matplotlib 1.2 and HoloViews 1.6 toolboxes. We implemented NMF and ICA with the Python scikit-learn NMF and fastica functions respectively^32^.

In addition to the separation algorithm, the FISSA package has two utilities, which may be used independently. First, FISSA has fast TIFF reading scripts, using the open source ${\mathtt{tifffile}}$ package. Second, FISSA has a baseline estimator which estimates *f*_0_ as the 5^th^ percentile of the signal after applying a 1 Hz low-pass filter.

### Simulated data

**Figure 6:**
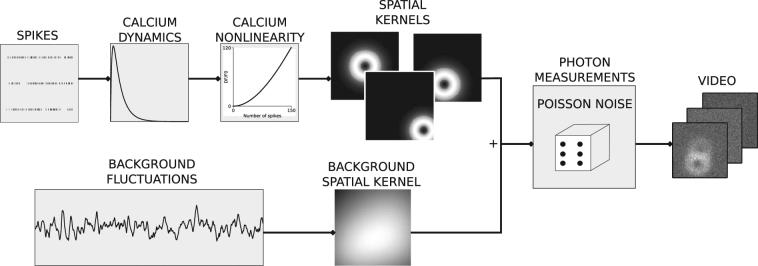
Simulated data generation. For each simulated neuron, a Poisson spike train is generated. The corresponding calcium indicator dynamics are simulated using GCaMP6 rise and decay times, and a nonlinearity. Next, the calcium traces are associated with a spatial kernel consisting of a doughnut shape mask (that models the cellular soma’s structure) and a Gaussian that simulates signal spread. Additionally, noisy background fluctuations are generated which vary spatially through a spatial kernel. All resulting signals are summed, and passed through a Poisson generation process to simulate photon emission, resulting in the final image sequence.

The simulated data generation is illustrated in Fig. 6. Each neuron spike train, *s*(*t*), is generated by a Poisson process at a given rate. The calcium dynamics for a given cell are modelled as a difference of exponentials

8 $$\begin{array}{rcl}\frac{d{c}_{d}}{dt}(t) & = & -\frac{1}{{\tau }_{d}}{c}_{d}(t)+s(t)\\ \frac{d{c}_{r}}{dt}(t) & = & -\frac{1}{{\tau }_{r}}{c}_{r}(t)+s(t)\\ c(t) & = & {c}_{d}(t)-{c}_{r}(t)\end{array}$$

where *c*_*d*_(*t*) and *c*_*r*_(*t*) model the decay and rise dynamics respectively, *c*(*t*) the overall calcium dynamics, and *τ*_*r*_ and *τ*_*d*_ the rise and decay time constants respectively. Using the published model of GCaMP6 dynamics^35,36^, a polynomial non-linearity to model calcium indicator is applied to obtain the measured calcium indicator signal

9 $$\begin{array}{l}d(t)=\,{\rm{\min }}\,[{c}_{{\mathtt{\max }}},c(t)]\\ f(t)=A\{d(t)+{p}_{2}[d{(t)}^{2}-d(t)]+{p}_{3}[d{(t)}^{3}-d(t)]\}\end{array}$$

where *A* sets the signal change corresponding to a single spike, and *p*_2_ and *p*_3_ are polynomial parameters. The saturation is applied for $c > {c}_{{\mathtt{\max }}}$, with ${{c}}_{{\mathtt{\max }}}=\frac{-2{p}_{2}-\sqrt{4{p}_{2}^{2}+12{p}_{3}({p}_{2}+{p}_{3}-\mathrm{1)}}}{6{p}_{3}}$; otherwise the model starts reducing the output fluorescence beyond ${c}_{{\mathtt{\max }}}$. Using the average values from^36^ we set *p*_2_ = 0.85, *p*_3_ = −0.006, *τ*_*r*_ = 0.0156 *s*, and *τ*_*d*_ = 0.76 *s* for model GCaMP6f dynamics, and *p*_2_ = 0.81, *p*_3_ = −0.056, *τ*_*r*_ = 0.0702 *s*, and *τ*_*d*_ = 1.87 *s* for GCaMP6s dynamics. *A* was set to 0.3%, 2%, and 4% for the central cell, the overlapping cell (Fig. 2C), and the bright localised signal (Fig. 2C) respectively. For Fig. 2 the simulations ran for 120 seconds at 100 Hz. The firing rates were set at 0.5 Hz, and 0.3 Hz for the central cell and the neighbouring cells, respectively (unless otherwise noted). In order to model the effects of stimulus presentation, and to induce correlations between neurons, all firing rates were periodically doubled for a duration of 15 seconds. For predicting calcium traces from the recorded spikes for Fig. 4, the simulations ran at 60 Hz for as long as each original neuron was recorded electrophysiologically, with the electrophysiologically measured spike-times binned at 60 Hz.

This signal *f*(*t*) is convolved with a two-dimensional doughnut-shaped spatial kernel given by the difference between two 2D Gaussians plus a step function

10 $${K}_{i}(x,y)=(\begin{array}{cc}c+S({\boldsymbol{\mu }},{\boldsymbol{\Sigma }})-{\mathscr{S}}({\boldsymbol{\mu }},{\boldsymbol{\Sigma }}/2), & {\rm{i}}{\rm{f}}\,{\mathscr{S}}({\boldsymbol{\mu }},{\boldsymbol{\Sigma }})-{\mathscr{S}}({\boldsymbol{\mu }},{\boldsymbol{\Sigma }}/2) > T\\ S({\boldsymbol{\mu }},{\boldsymbol{\Sigma }})-{\mathscr{S}}({\boldsymbol{\mu }},{\boldsymbol{\Sigma }}/2), & \,{\rm{o}}{\rm{t}}{\rm{h}}{\rm{e}}{\rm{r}}{\rm{w}}{\rm{i}}{\rm{s}}{\rm{e}}\,\end{array}$$

where ${\mathscr{S}}({\boldsymbol{\mu }},{\boldsymbol{\Sigma }})=\exp (-\frac{1}{2}{({\bf{x}}-{\boldsymbol{\mu }})}^{T}\,{{\boldsymbol{\Sigma }}}^{-1}({\bf{x}}-{\boldsymbol{\mu }}))$, ***μ*** = [*μ*_*x*_, *μ*_*y*_] indicates the *x* and *y* positions, and $${\boldsymbol{\Sigma }}=[\begin{array}{cc}{\sigma }^{2} & \rho \\ \rho  & {\sigma }^{2}\end{array}]$$ sets the spatial spread. The offset *c* = 0.2 models the physical structure of a cell soma, the threshold *T* = 0.5 determines its extent, and the Gaussian models the spread of a cell’s calcium signal beyond its structure to model cross contamination between nearby structures. *K*_*i*_(*x*, *y*) was additionally normalized so that ${\mathtt{\max }}(K)=1$. For Fig. 2 the central cell had *σ*^2^ = 50 and *μ*_*x*_ = *μ*_*y*_ = 0 (indicating the middle of the field of view), the overlapping cell had *σ*^2^ = 50 and *μ*_*x*_ = *μ*_*y*_ = 13, and the small cell *σ*^2^ = 10 and *μ*_*x*_ = *μ*_*y*_ = −15. Unless otherwise noted *ρ* is always set to 0.

We simulate the background neuropil contamination *B*(*t*) as

11 $$\frac{dB(t)}{dt}=\eta \,dW(t),$$

where *η* is a constant (set to 0.05) and *W*(*t*) is a Wiener process. A square wave signal of magnitude 0.1 and a period of 15 seconds was also added to simulate stimulus presentation. To vary the background signal spatially, the background signal is convolved with a spatial kernel given by the sum of *M* = 10 Gaussians

12 $${K}_{{\mathtt{bg}}}(x,y)=\sum _{i=1}^{M}\,\exp \,(-\frac{{(x-{\mu }_{xi})}^{2}+{(y-{\mu }_{yi})}^{2}}{2{\sigma }_{i}^{2}})\mathrm{.}$$

with ${\sigma }_{i}^{2}\in \mathrm{[100},\mathrm{200]}$ and *μ*_*xi*_, *μ*_*yi*_ give the mean *x* and *y* positions which were randomly drawn within the image limits (80 by 80 pixels). Finally, we obtained the calcium response at a given pixel at position *x*, *y* and time *t* as

13 $$F(x,y,t)=\sum _{i}\,{K}_{i}(x,y){f}_{{\mathtt{true}},i}(t)+{K}_{{\mathtt{bg}}}(x,y)B(t),$$

where the sum over *i* is across structures. To simulate photon emission, *F* is used as the rate in a Poisson noise process to generate the final pixel responses. Signals for each ROI are estimated based on the mask

14 $${M}_{i}(x,y)=(\begin{array}{cc}1, & {\rm{i}}{\rm{f}}\,{K}_{i}(x,y) > {T}_{{\mathtt{m}}{\mathtt{a}}{\mathtt{s}}{\mathtt{k}}}\\ 0, & \,{\rm{o}}{\rm{t}}{\rm{h}}{\rm{e}}{\rm{r}}{\rm{w}}{\rm{i}}{\rm{s}}{\rm{e}}\,\end{array},$$

where ${T}_{{\mathtt{mask}}}$ determines its extent (set to 0.5 unless otherwise noted).

### Other decontamination methods used to compare FISSA performance

#### Neuropil subtraction

Given a ROI’s spatially averaged signal ${f}_{{\mathtt{ROI}}}(t)$ and a surrounding neuropil region, the neuropil’s spatially averaged signal ${f}_{{\mathtt{npil}}}(t)$ was subtracted to give the estimated signal

15 $${f}_{{\mathtt{est}}}(t)={f}_{{\mathtt{ROI}}}(t)-k\,{f}_{{\mathtt{npil}}}(t),$$

where *k* is a constant. The value of this constant has been determined manually in previous publications^3,14^. When analysing our simulated data, we set *k* = 1, since this value removed the background fluctuations (Fig. 2A). For the experimental *in vivo* data, we set *k* = 0.7, as previously published^3^. For the neuropil region we used the total surrounding region (all subregions) as defined by the FISSA algorithm.

#### Constrained non-negative matrix factorization

For details of the cNMF algorithm see the original publication^9^. We applied cNMF to the simulated (Fig. 2) dataset using the $\mathrm{demo}{\mathtt{\_}}\mathrm{script}{\mathtt{.m}}$ script from the cNMF toolbox. For the *in vivo* data presented in Figs 4 and 5 the memory usage was much higher than for the simulated dataset due to higher resolution field of views and more frames. We thus performed the analysis in patches, rather than analysing the whole field of view. For this, we used the $\mathrm{run}{\mathtt{\_}}\mathrm{pipeline}{\mathtt{.m}}$ script from the cNMF toolbox. cNMF often detected more regions than just the soma of interest. In these cases we chose the region that resulted in the highest performance (highest Pearson correlation values for all subpanels (iii) in Figs 2 and 4B).

### *In vivo* calcium imaging data

All procedures were performed in accordance with the animal care and handling guidelines of the University of Edinburgh animal welfare committee, and were performed under a UK Home Office project license.

For the data in Fig. 5, all surgical and imaging procedures are detailed in our previous publication^4^. Briefly, adeno-associated viruses (AAV1.Syn.GCaMP6f.WPRE.SV40, University of Pennsylvania Vector Core, PA, USA) were locally injected in V1 at three different depths (−50, −400, and −600 *μm*) in 8 mice (8- to 10-week-old). The mice were obtained by crossing Cre-driver transgenic mice lines (Sst<tm2.1(cre)Zjh> [RRID:IMSR_JAX:013044] n = 3, Pvalb<tm1(cre)Arbr> (PV-Cre) [RRID:IMSR_JAX:008069] n = 1, or Vip<tm1(cre)Zjh> [RRID:IMSR_JAX:010908] n = 4) with Rosa-CAG-LSL-tdTomato [RRID:IMSR_JAX:007914] mice. All mice were originally obtained from Jackson Laboratory, ME, USA. Mice were group housed (typically 2–4 mice) and both male and female mice were used for the experiments. After 2–3 weeks of expression, the running speed and GCaMP6f signals were simultaneously recorded, during the presentation of drifting gratings (the total visual stimulation times were 720–1200 seconds per imaged field of view^4^). For the results presented in Fig. 5, we did not exclude tdTomato-positive neurons such that all GCaMP6f labelled neurons were included in the analysis.

#### Motion correction

To perform motion correction for the *in vivo* data presented in Figs 4 and 5 we used the discrete Fourier method from the SIMA toolbox^8^.

### Data availability statement

The toolbox described in this paper is available at https://github.com/rochefort-lab/fissa.

## Electronic supplementary material

Supplemenentary information

## References

[1] Huber D (2012). Multiple dynamic representations in the motor cortex during sensorimotor learning. Nature.

[2] Margolis DJ (2012). Reorganization of cortical population activity imaged throughout long-term sensory deprivation. Nat Neurosci.

[3] Chen T (2013). Ultrasensitive fluorescent proteins for imaging neuronal activity. Nature.

[4] Pakan J (2016). Behavioural state modulation of inhibition is context-dependent and cell-type specific in mouse V1. Elife.

[5] Attinger A, Wang B, Keller G (2017). Visuomotor coupling shapes the functional development of mouse visual cortex. Cell.

[6] Dombeck D, Khabaz A, Collman F, Adelman T, Tank D (2007). Imaging large-scale neural activity with cellular resolution in awake, mobile mice. Neuron.

[7] Greenberg D, Kerr J (2009). Automated correction of fast motion artifacts for two-photon imaging of awake animals. Journal of Neuroscience Methods.

[8] Kaifosh, P., Zaremba, J. D., Danielson, N. B. & Losonczy, A. SIMA: Python software for analysis of dynamic fluorescence imaging data. *Frontiers in neuroinformatics* **8** (2014). 10.3389/fninf.2014.00080 PMC4172099 25295002

[9] Pnevmatikakis EA (2015). Simultaneous Denoising, Deconvolution, and Demixing of Calcium Imaging Data. Neuron.

[10] Muir, D., Roth, M., Helmchen, F. & Kampa, B. Model-based analysis of pattern motion processing in mouse primary visual cortex. *Frontiers in neural circuits* **9** (2015). 10.3389/fncir.2015.00038 PMC4525018 26300738

[11] Pachitariu, M. *et al*. Suite2p: beyond 10,000 neurons with standard two-photon microscopy. *bioRxiv*, 10.1101/061507 (2016).

[12] Dubbs, A., Guevara, J. & Yuste, R. moco: Fast motion correction for calcium imaging. *Frontiers in Neuroinformatics* **10** (2016). 10.3389/fninf.2016.00006 PMC4754735 26909035

[13] Pnevmatikakis, E. & Giovannucci, A. Normcorre: An online algorithm for piecewise rigid motion correction of calcium imaging data. *bioRxiv*, 10.1101/108514 (2017). 10.1016/j.jneumeth.2017.07.031 28782629

[14] Peron S, Freeman J, Iyer V, Guo C, Svoboda K (2015). A cellular resolution map of barrel cortex activity during tactile behavior. Neuron.

[15] Maruyama R (2014). Detecting cells using non-negative matrix factorization on calcium imaging data. Neural Networks.

[16] Diego F, Hamprecht F (2014). Sparse space-time deconvolution for calcium image analysis. NIPS.

[17] Apthorpe, N. *et al*. Automatic neuron detection in calcium imaging data using convolutional networks. *NIPS* **29** (2016).

[18] Mukamel EA, Nimmerjahn A, Schnitzer MJ (2009). Automated Analysis of Cellular Signals from Large-Scale Calcium Imaging Data. Neuron.

[19] Peron S, Chen T, Svoboda K (2015). Comprehensive imaging of cortical networks. Current opinion in neurobiology.

[20] Stetter M (2000). Principal component analysis and blind separation of sources for optical imaging of intrinsic signals. NeuroImage.

[21] Ji N, Sato T, Betzig E (2012). Characterization and adaptive optical correction of aberrations during *in vivo* imaging in the mouse cortex. PNAS.

[22] Cichocki A, Anh-Huy PHAN (2009). Fast local algorithms for large scale nonnegative matrix and tensor factorizations. IEICE transactions on fundamentals of electronics, communications and computer sciences.

[23] Langville, A. N., Meyer, C. D., Albright, R., Cox, J. & Duling, D. Algorithms, initializations, and convergence for the nonnegative matrix factorization. *arXiv* 1407.7299 (2014).

[24] Svoboda, H. K. Simultaneous imaging and loose-seal cell-attached electrical recordings from neurons expressing a variety of genetically encoded calcium indicators. *GENIE project, Janelia Farm Campus*, *CRCNS*.*org* (2015).

[25] Niell CM, Stryker MP (2010). Modulation of visual responses by behavioral state in mouse visual cortex. Neuron.

[26] Keller G, Bonhoeffer T, Hübener M (2012). Sensorimotor mismatch signals in primary visual cortex of the behaving mouse. Neuron.

[27] Ayaz A, Saleem A, Schölvink M, Carandini M (2013). Locomotion controls spatial integration in mouse visual cortex. Current Biology.

[28] Erisken S (2014). Effects of locomotion extend throughout the mouse early visual system. Current Biology.

[29] Fu Y (2014). A cortical circuit for gain control by behavioral state. Cell.

[30] Dadarlat M, Stryker M (2017). Locomotion enhances neural encoding of visual stimuli in mouse v1. Journal of Neuroscience.

[31] Friedrich, J. *et al*. Multi-scale approaches for high-speed imaging and analysis of large neural populations. *PLoS Comput Biol* **13** (2017). 10.1371/journal.pcbi.1005685 PMC5557609 28771570

[32] Pedregosa F (2011). Scikit-learn: Machine learning in Python. Journal of Machine Learning Research.

[33] Lin C (2007). Projected gradient methods for non-negative matrix factorization. Neural Computation.

[34] Boutsidis C, Gallopoulos E (2008). Svd based initialization: A head start for nonnegative matrix factorization. Pattern Recognition.

[35] Akerboom J (2012). Optimization of a GCaMP Calcium Indicator for Neural Activity Imaging. The Journal of Neuroscience.

[36] Deneux, T. *et al*. Accurate spike estimation from noisy calcium signals for ultrafast three-dimensional imaging of large neuronal populations *in vivo*. *Nature Communications* **7** (2016). 10.1038/ncomms12190 PMC4960309 27432255

