# Supplementary material for: FISSA: A neuropil decontamination toolbox for calcium imaging signals
Source: Sci Rep. 2018 Feb 22;8:3493. doi: 10.1038/s41598-018-21640-2 (PMC5823956; doi:10.1038/s41598-018-21640-2)
Supplement: Supplementary file 1 — Supplemenentary information [file 41598_2018_21640_MOESM1_ESM.pdf]

SUPPLEMENT - FISSA: A neuropil decontamination toolbox for calcium imaging signals

Sander W. Keemink[1,2ς*], Scott C. Lowe[1,3ς], Janelle M.P. Pakan[3], Evelyn Dylda[3], Mark C.W. van Rossum[1†] and Nathalie L. Rochefort[3†*]

13th February 2018

[1]Institute for Adaptive and Neural Computation, School of Informatics, University of Edinburgh, Edinburgh EH8 9AB, UK
[2]Bernstein Center Freiburg, Faculty of Biology, University of Freiburg, 79104 Freiburg, Germany
[3]Centre for Discovery Brain Sciences, Biomedical Sciences, University of Edinburgh, Edinburgh, EH8 9XD, UK

*corresponding authors: swkeemink@scimail.eu, n.rochefort@ed.ac.uk
ςShared first authors
†Shared last authors

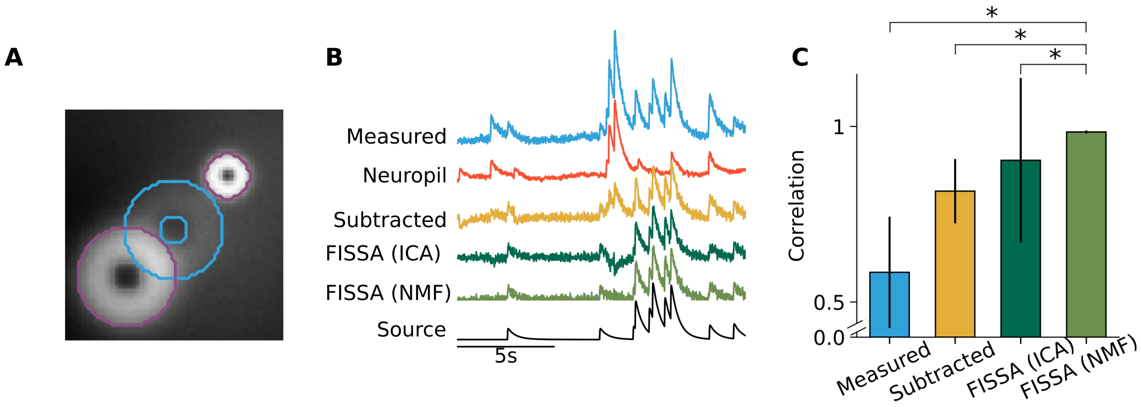

Supplementary Figure 1: Comparison of the NMF method used in FISSA to the equivalent ICA method. Same example case and legend as Fig. 2C. Results obtained with FISSA using ICA are shown in dark green. Occasionally ICA can lead to negative signals (as shown in panel B) leading to a larger variance in performance. Error bars indicate standard deviation. *: p<0.05; Wilcoxon signed-rank test, $n = 10$ simulations of 120 s each. FISSA (NMF) vs: measured $p = 0.0051$, subtraction $p = 0.0051$, FISSA (ICA) $p = 0.0051$.
